# WE'RE ALL MAKERS NOW: ARTS AND CRAFT PARTICIPATION IN MIDLIFE

**DOI:** 10.1093/geroni/igac059.845

**Published:** 2022-12-20

**Authors:** Carolyn Adams-Price

**Affiliations:** Mississippi State University, STARKVILLE, Mississippi, United States

## Abstract

Recent research has focused on the benefits of participation in creative activities for older adults, even those who experiencing cognitive deficits. However, many people begin participating in creative activities in midlife, and continue their participation into later life. Research in my lab suggests that middle aged people turn to crafts that involve making things to reduce stress and increase feelings of productivity. Glaveanu’s sociocultural theory will be introduced to explain the benefits middle aged people get from creating crafts, and how those benefits are accrued from the beginner stage through the development of expertise.

